# *KLB* gene polymorphism is associated with obesity and non-alcoholic fatty liver disease in the Han Chinese

**DOI:** 10.18632/aging.102293

**Published:** 2019-09-23

**Authors:** Fang Ji, Ye Liu, Jun-Gui Hao, Li-Ping Wang, Ming-Jia Dai, Gui-Fang Shen, Xue-Bing Yan

**Affiliations:** 1 Department of Infectious Diseases, Nanjing Medical University, Nanjing, Jiangsu, China; 2 Department of Infectious Disease, The Affiliated Hospital of Xuzhou Medical University, Xuzhou, Jiangsu, China; 3 Department of Infectious Disease, Xuzhou Medical University, Xuzhou, Jiangsu, China; 4 Department of Physical Examination Center, The Affiliated Hospital of Xuzhou Medical University, Xuzhou, Jiangsu, China

**Keywords:** Klotho beta, fibroblast growth factor, single-nucleotide polymorphisms, non-alcoholic fatty liver disease, obesity

## Abstract

Klotho beta (KLB) mediates binding of fibroblast growth factor (FGF) 21 to the FGF receptor (FGFR). FGF21-KLB-FGFR signaling regulates multiple metabolic systems in the liver, and we hypothesized that *FGF21*, *KLB* and *FGFR* single-nucleotide polymorphisms (SNPs) are involved in hepatic lipid accumulation. The SNPs were detected in 1688 individuals divided into four groups: non-obese without non-alcoholic fatty liver disease (NAFLD), obese without NAFLD, non-obese with NAFLD, and obese with NAFLD. The A-allele of *KLB* SNP rs7670903 correlated with higher body mass index (*P* = 0.0005), and the A-allele frequency was higher in the obese than non-obese group (*P* = 0.003). The G-allele frequency of *KLB* rs7674434 and T-allele frequency of rs12152703 were higher in the obese with NAFLD than obese without NAFLD group (*P* = 0.004 and *P* = 0.006), but the genotype distribution between two non-obese groups did not differ. *KLB* rs7674434 and rs12152703 had associations with alanine aminotransferase (ALT) (*P* = 0.03 and *P* = 0.04, respectively) and gamma-glutamyltransferase (*P* = 0.03 and *P* = 0.02, respectively) levels in all subjects, but the associations were especially strong with ALT in the NAFLD group (*P* = 0.005 and *P* = 0.008, respectively). These findings suggest that *KLB* SNPs are related to obesity and hepatic inflammation and that they may be involved in the pathogenesis of NAFLD.

## INTRODUCTION

The klotho family protein klotho beta (KLB) is a single transmembrane protein that serves as a co-receptor with fibroblast growth factor receptor (FGFR) [1]. By mediating FGF21 binding to FGFR, KLB plays key roles in the regulation of metabolism [2]. Under fasting and some stress conditions, expression of FGF21 rises in the liver, adipose tissue, pancreas and brain [3, 4], where signaling via the FGF21-KLB-FGFR1 complex exerts effects impacting multiple metabolic systems [5].

Obesity and non-alcoholic fatty liver disease (NAFLD) are two crucial health problems worldwide. NAFLD is now thought to be a hepatic manifestation of metabolic syndrome caused by an underlying disorder of energy utilization and storage [6]. Normally, FGF21 mediates energy expenditure that causes weight loss and a reduction in hepatic lipidosis [7]. But in obesity patients, the serum FGF21 levels are elevated. It has therefore been hypothesized that obesity leads to a FGF21-resistant state. In mice, moreover, decreased *KLB* expression appears to result in FGF21 resistance [8]. This suggests FGF21-KLB-FGFR signaling may be involved in the pathogenesis of NAFLD.

The development of hepatic steatosis involves the interaction of genetic and environmental factors [6]. Several studies suggest that genetic variants of *FGF21* associate with nutrient intake and metabolism [9, 10]. Studies in obese mice verified that *KLB* expression is associated with lipid homeostasis and FGF21 resistance [8, 11, 12]. In that regard, little has been reported about the correlation between *FGF21* gene polymorphisms and NAFLD, and there have been no reports on the relationship between *KLB* gene polymorphism and NAFLD. We hypothesized that single nucleotide polymorphisms (SNPs) in *FGF21*, *KLB* and *FGFR* are involved in hepatic lipid accumulation and the pathogenesis of NAFLD.

## RESULTS

### Study population

Among the 1688 participants, the frequencies of NAFLD and obesity were 32.29% and 37.44%, respectively. Comparison of the clinical and biochemical characteristics of the subjects revealed no significant difference between the NAFLD and non-NAFLD groups (Table 2). By contrast, the baseline characteristics of the obese and non-obese groups differed significantly (Table 3). Compared to the non-obese group, subjects in obese group were older and had a higher male-to-female ratio; higher fasting glucose and serum lipid levels; and higher alanine aminotransferase (ALT), aspartate aminotransferase (AST), and gamma-glutamyltransferase (γ-GT) levels. All samples were successfully genotyped for SNPs.

### Quality control of candidate SNPs

The positions of all the SNPs analyzed are listed in Table 1. Of those, two, rs838133 (*FGF21*) and rs2071616 (*FGFR2*), which had minor allele frequencies (MAFs) < 10% were eliminated. The loss frequency for the genotyping was less than 0.1%. Deviation of the SNPs from Hardy-Weinberg equilibrium (HWE) was tested in each population using Fisher’s exact test. The SNP rs7012413 (*FGFR2*) was the only one that deviated from HWE (*P <* 0.05); the allele frequencies of all other SNPs reached HWE. The genotypes and allele frequencies of the SNPs are listed in Table 4.

**Table 1:** Information on the SNPs and primers.

**Gene**	**Chr.**	**Position**	**SNP**	**EXT1-SEQ**	**EXT2-SEQ**
*KLB*	4	39441340	rs7670903	aaacAAAGACATTTTTGGAGCAC	aaacAAAGACATTTTTGGAGCAT
*KLB*	4	39417789	rs7674434	GGTACCTGTAATATATTATCTCATG	GGTACCTGTAATATATTATCTCATT
*KLB*	4	39420199	rs12152703	gGGATCTTTTAGTATTTGGGGG	gGGATCTTTTAGTATTTGGGGT
*KLB*	4	39429811	rs2608819	TTGCCAAGTAACAAGGTAAAAAA	TTGCCAAGTAACAAGGTAAAAAG
*FGF21*	19	48763133	rs499765	TTGCAAAGGATTTGGGATC	TTGCAAAGGATTTGGGATG
*FUT1*	19	48753131	rs838136	cccaCTGGCAAACAGGTTCTCCA	cccaCTGGCAAACAGGTTCTCCG
*FGFR1*	8	38461831	rs4733946	CAAATGTAAAGAATTATGCATTTGG	CAAATGTAAAGAATTATGCATTTGT
*FGFR2*	10	121516577	rs3135761	TCTGCCTCTTTGTCATTTC	TCTGCCTCTTTGTCATTTT

SNP: single nucleotide polymorphism, Chr: chromosome, EXT1-SEQ: extention primer sequence of allele 1, EXT2-SEQ: extension primer sequence of allele 2.

**Table 2:** General clinical characteristics of the NAFLD and non-NAFLD cohorts.

**Characteristic**	**NAFLD subjects (n=545)**	**Non-NAFLD controls (n=1143)**	***P***	**NAFLD subjects**			**Non-NAFLD controls**		
				**Obesity (n=202)**	**Non-obesity (n=343)**	***p***	**Obesity (n=382)**	**Non-obesity (n=761)**	***p***
Age, years	42.39±12.60	42.22±12.52	0.862	44.76±31.13	40.35±11.87	<0.001	44.83±12.96	40.83±12.19	<0.001
Male/female	218/327	487/656	0.297	106/96	127/216	<0.001	223/159	256/505	<0.001
BMI, kg/m2	24.11±3.51	23.99±3.33	0.548	27.56±2.49	21.97±2.01	<0.001	27.56±2.35	22.12±1.97	<0.001
ALT, U/L	23.78±17.59	23.67±25.52	0.261	31.34±22.65	19.23±11.82	<0.001	31.01±36.84	19.97±16.26	<0.001
AST, U/L	25.18±14.51	21.25±9.72	0.437	35.39±11.68	20.54±5.99	<0.001	25.06±15.81	21.56±10.97	<0.001
γ-GT, U/L	24.95±20.91	25.46±27.33	0.502	31.79±23.68	20.54±17.49	<0.001	35.46±36.72	20.48±19.48	<0.001
Serum triglyceride, mmol/L	1.39±1.11	1.31±1.03	0.639	1.74±1.29	1.68±0.91	<0.001	1.68±1.43	1.13±0.68	<0.001
Serum total cholesterol, mmol/L	4.90±1.01	4.87±1.00	0.951	5.05±1.06	4.81±0.98	<0.001	6.52±1.48	4.80±0.93	<0.001
Serum LDL-C, mmol/L	2.85±0.67	2.84±0.69	0.895	3.01±0.70	2.75±0.62	<0.001	2.99±0.68	2.77<±0.66	<0.001
Fasting plasma glucose, mmol/L	5.32±1.06	5.34±1.03	0.532	5.58±1.16	5.17±1.00	<0.001	5.70±1.41	5.15±0.71	<0.001
Serum FGF21, pg/ml	293.07±258.03	303.42±291.79	0.921	361.35±306.29	244.70±205.25	<0.001	366.63±311.56	267.57±269.94	0.003

Data are means±SD or counts. *P*: level of statistical significance with the Kruscal-Wallis non-parametric test.

**Table 3:** General clinical characteristics of the obese and non-obese cohorts.

**Characteristic**	**Obese subjects (n=584)**	**Non-obese subjects (n=1104)**	***P***	**Obese subjects**			**Non-obese subjects**		
				**NAFLD (n=202)**	**Non-NAFLD (n=382)**	***P***	**NAFLD (n=343)**	**Non-NAFLD (n=761)**	***P***
Age, years	42.39±12.60	42.22±12.52	<0.001	44.76±31.13	44.83±12.96	0.559	40.35±11.87	40.83±12.19	0.516
Male/female	218/327	487/656	<0.001	106/96	223/159	0.142	127/216	256/505	0.554
BMI, kg/m2	24.11±3.51	23.99±3.33	<0.001	27.56±2.49	27.56±2.35	0.736	21.97±2.01	22.12±1.97	0.224
ALT, U/L	23.78±17.59	23.67±25.52	<0.001	31.34±22.65	31.01±36.84	0.200	19.23±11.82	19.97±16.26	0.625
AST, U/L	25.18±14.51	21.25±9.72	<0.001	35.39±11.68	25.06±15.81	0.052	20.54±5.99	21.56±10.97	0.296
γ-GT, U/L	24.95±20.91	25.46±27.33	<0.001	31.79±23.68	35.46±36.72	0.635	20.54±17.49	20.48±19.48	0.933
Serum triglyceride, mmol/L	1.39±1.11	1.31±1.03	<0.001	1.74±1.29	1.68±1.43	0.587	1.68±0.91	1.13±0.68	0.479
Serum total cholesterol, mmol/L	4.90±1.01	4.87±1.00	<0.001	5.05±1.06	6.52±1.48	0.838	4.81±0.98	4.80±0.93	0.790
Serum LDL-C, mmol/L	2.85±0.67	2.84±0.69	<0.001	3.01±0.70	2.99±0.68	0.895	2.75±0.62	2.77±0.66	0.608
Fasting plasma glucose, mmol/L	5.32±1.06	5.34±1.03	<0.001	5.58±1.16	5.70±1.41	0.510	5.17±1.00	5.15±0.71	0.278
Serum FGF21, pg/ml	293.07±258.03	303.42±291.79	<0.001	361.35±306.29	366.63±311.56	0.924	244.70±205.25	267.57±269.94	0.605

Data are means±SD or counts. *P*: level of statistical significance with the Kruscal-Wallis non-parametric test.

**Table 4:** Genotype and allele frequencies of the SNPs.

**Gene**	**SNP**	**Call rate (%)**	**Minor allele**	**Major allele**	**MAF cases**	**MAF controls**	**HWE controls**
*KLB*	rs7670903	99.4	A	G	0.52	0.49	0.35
*KLB*	rs7674434	99.6	G	T	0.13	0.12	0.56
*KLB*	rs12152703	99.4	T	G	0.13	0.12	0.43
*KLB*	rs2608819	99.2	T	C	0.18	0.16	0.11
*FGF21*	rs499765	99.0	G	C	0.45	0.45	0.15
*FUT1*	rs838136	99.8	C	T	0.37	0.36	0.27
*FGFR1*	rs4733946	99.1	T	G	0.12	0.12	0.58
*FGFR2*	rs3135761	99.2	A	G	0.48	0.48	0.33

Abbreviations: MAF: minor allele frequency, HWE: Hardy-Weinberg equilibrium test.

### Association between genotypes and obesity

**Figure 1:**
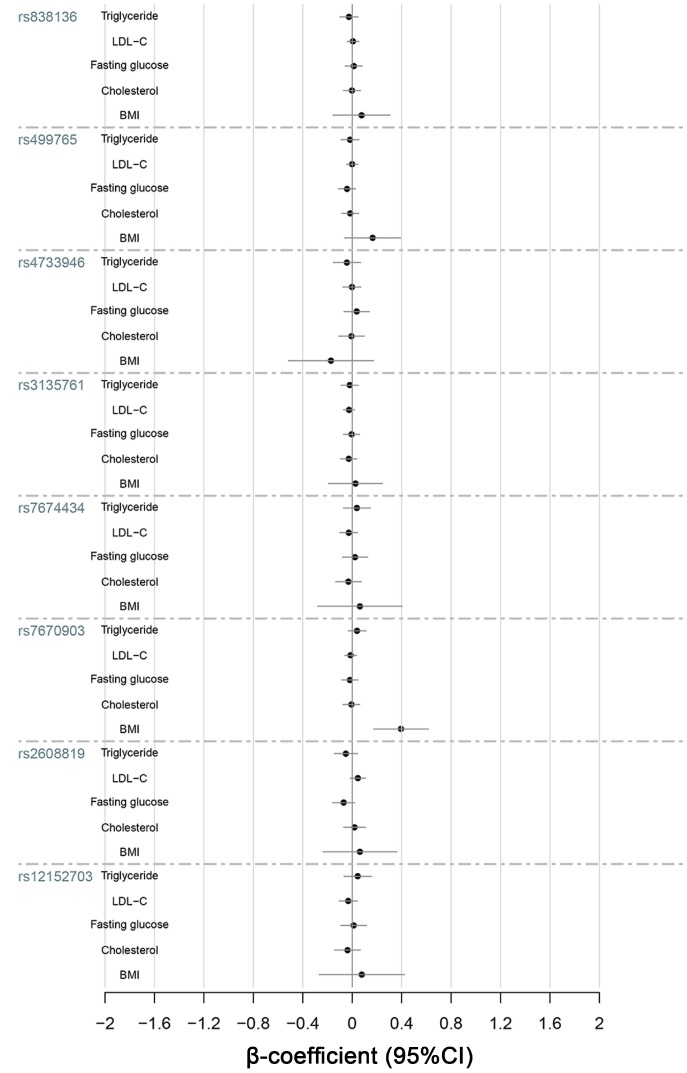
**Quantitative-trait associations between triglycerides, LDL-C, fasting glucose, cholesterol and BMI and the alleles of KLB, FGFR1 and FGF21 were analyzed for all 1688 subjects using multiple linear regression adjusted for sex and age.**

The results of quantitative-traits association analyses adjusted for gender and age (Figure 1) indicated that SNP rs7670903 in *KLB* had a significant association with BMI (*P* = 0.0005) (Table 5). The minor A-allele of rs7670903 correlated with a higher BMI. When we compared the allele frequencies between the obese and non-obese groups using the dominant and additive model covariate adjusted association analyses (Table 6), the rs7670903 A-allele frequency was significantly higher in obese group than the non-obese group in both models (*P* = 0.003 and *P* = 0.011, respectively).

**Table 5:** Significant associations between SNPs and NAFLD phenotypes in a covariate adjusted linear regression analysis.

**Trait**	**Gene**	**SNP**	**Risk allele**	**β-coefficient**	**STAT**	***P***
BMI	*KLB*	rs7670903	A	0.40	3.49	<0.001
ALT	*KLB*	rs7674434	G	2.59	2.17	0.030
		rs12152703	T	2.47	2.04	0.041
γ-GT	*KLB*	rs7674434	G	2.77	2.15	0.032
		rs12152703	T	2.94	2.26	0.024
		rs7670903	A	1.78	2.12	0.034
AST/ALT	*KLB*	rs7674434	G	0.06	2.26	0.024
		rs12152703	T	0.05	2.47	0.013
FGF21	*FGF21*	rs499765	G	19.35	1.97	0.049

β-coefficient: estimated quantitative effect of each SNP minor allele copy on a phenotype of NAFLD, STAT: T statistic, *P*: level of statistical significance of the covariate adjusted linear regression analysis.

**Table 6:** Associations between SNPs and obesity.

**Gene**	**SNP**	**Minor allele**	**Dominate model**				**Additive model**		
			**OR (95%CI)**	**SE**	***P***		**OR (95%CI)**	**SE**	***P***
*KLB*	rs7670903	A	1.43 (1.13,1.81)	0.12	0.003		1.20 (1.04,1.40)	0.07	0.011
*KLB*	rs7674434	G	1.04 (0.82,1.34)	0.13	0.723		1.03 (0.82,1.29)	0.11	0.784
*KLB*	rs12152703	T	1.06 (0.83,1.36)	0.13	0.640		1.05 (0.84,1.32)	0.12	0.665
*KLB*	rs2608819	T	1.03 (0.82,1.28)	0.11	0.828		1.02 (0.84,1.24)	0.10	0.835
*FGF21*	rs499765	G	1.00 (0.80,1.25)	0.11	0.997		1.08 (0.93,1.25)	0.08	0.324
*FUT1*	rs838136	C	1.14 (0.93,1.41)	0.11	0.214		1.08 (0.93,1.07)	0.08	0.298
*FGFR1*	rs4733946	T	0.99 (0.78,1.25)	0.16	0.956		1.01 (0.81,1.26)	0.11	0.939
*FGFR2*	rs3135761	A	1.02 (0.81,1.28)	0.11	0.862		1.05 (0.91,1.21)	0.07	0.531

Abbreviations: OR: odds ratio, 95%CI: 95% confidence interval, SE: standard error, *P*: level of statistical significance of the difference between obesity cases and controls.

Dominate model: Dominate model covariate adjusted association analysis, adjusted for age (years) and sex.

Additive model: Additive model covariate adjusted association analysis, adjusted for age (years) and sex.

### Association between genotypes and NAFLD

Comparison of the genotype distribution between the NAFLD and non-NAFLD groups revealed that the rs7670903 A-allele frequency tended to be lower in the NAFLD group (Table 7), but covariate adjusted association analysis showed no significant difference (*p* = 0.064). More importantly, comparison of genotype distributions among all four groups (Table 8) revealed that the G-allele frequency of rs7674434 and T-allele frequency of rs12152703 in *KLB* were significantly higher in the obese with NAFLD group than in the obese without NAFLD group in both the dominant model (*p* = 0.004 and *p* = 0.006, respectively) and additive model (*p* = 0.005 and *p* = 0.007, respectively) covariate adjusted association analysis. By contrast, there were no difference in the genotype distributions between with non-obese with NAFLD and without NAFLD groups.

**Table 7:** Associations between SNPs and NAFLD.

**Gene**	**SNP**	**Minor allele**	**Dominate model**		
			**OR (95%CI)**	**SE**	***P***
*KLB*	rs7670903	A	0.75 (0.55,1.02)	0.16	0.064
*KLB*	rs7674434	G	1.07 (0.78,1.46)	0.16	0.680
*KLB*	rs12152703	T	1.05 (0.77,1.44)	0.16	0.757
*KLB*	rs2608819	T	1.09 (0.82,1.45)	0.15	0.567
*FGF21*	rs499765	G	0.95 (0.71,1.26)	0.15	0.718
*FUT1*	rs838136	C	0.81 (0.62,1.06)	0.14	0.124
*FGFR1*	rs4733946	T	0.91 (0.66,1.24)	0.16	0.535
*FGFR2*	rs3135761	A	0.889 (0.66,1.17)	0.15	0.379

Abbreviations: OR: odds ratio, 95%CI: 95% confidence interval, SE: standard error, *P*: level of statistical significance of the difference between NAFLD cases and controls.

Dominate model: Additive model covariate adjusted association analysis, adjusted for age (years), sex and BMI (kg/m2).

**Table 8:** Associations between SNPs and NAFLD in the obese group.

**Gene**	**SNP**	**Minor allele**	**Dominate model**				**Additive model**		
			**OR (95%CI)**	**SE**	***P***		**OR (95%CI)**	**SE**	***P***
*KLB*	rs7670903	A	1.03 (0.69,1.53)	0.20	0.881		0.98 (0.75,1.27)	0.13	0.862
*KLB*	rs7674434	G	1.89 (1.22,2.93)	0.22	0.004		1.78 (1.19,2.67)	0.21	0.005
*KLB*	rs12152703	T	1.86 (1.20,2.88)	0.22	0.006		1.75 (1.16,2.63)	0.21	0.007
*KLB*	rs2608819	T	1.30 (0.88,1.22)	0.20	0.191		1.28 (0.91,1.82)	0.18	0.160
*FGF21*	rs499765	G	1.12 (0.76,1.63)	0.19	0.572		1.14 (0.89,1.46)	0.13	0.302
*FUT1*	rs838136	C	0.69 (0.47,0.99)	0.19	0.045		0.82 (0.64,1.06)	0.13	0.135
*FGFR1*	rs4733946	T	1.10 (0.72,1.69)	0.22	0.647		1.10 (0.75,1.61)	0.19	0.624
*FGFR2*	rs3135761	A	1.03 (0.70,1.52)	0.20	0.87		1.06 (0.83,1.35)	0.12	0.631

Abbreviations: OR: odds ratio, 95%CI: 95% confidence interval, SE: standard error, *P*: level of statistical significance of the difference between the obesity cases and controls.

Dominate model: Dominate model covariate adjusted association analysis, adjusted for age (years) and sex.

Additive model: Additive model covariate adjusted association analysis, adjusted for age (years) and sex.

### Association between genotypes and quantitative-traits related to NAFLD

**Figure 2:**
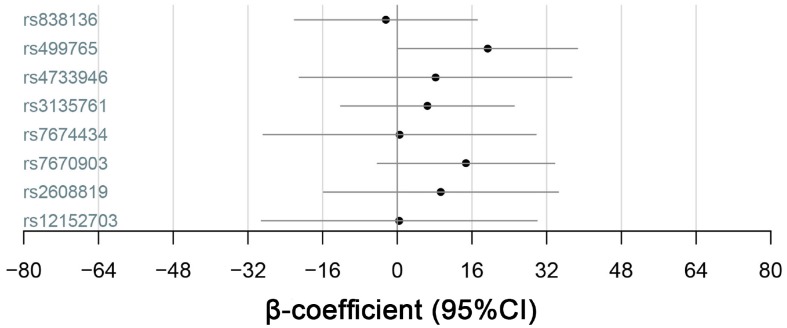
**Quantitative-trait associations between serum FGF21 levels and the alleles of KLB, FGFR1 and FGF21 were analyzed for all 1688 subjects using multiple linear regression adjusted for sex and age.**

**Figure 3:**
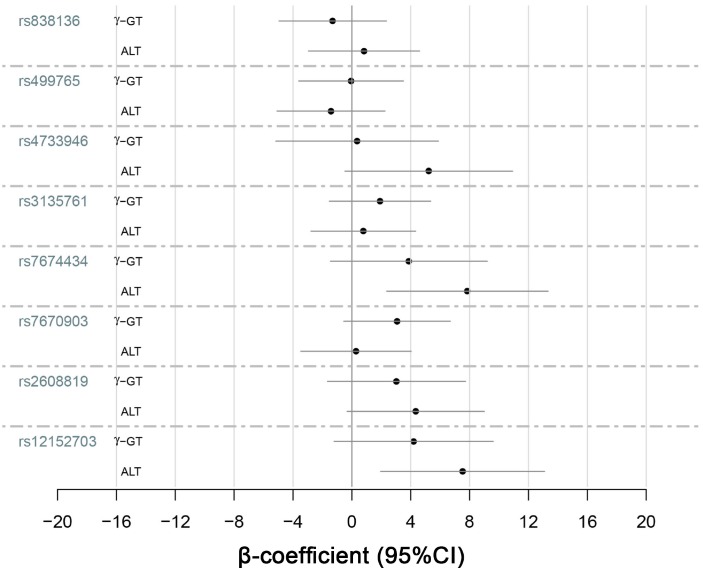
**Quantitative-trait associations between γ-GT and ALT levels and the alleles of KLB, FGFR1 and FGF21 were analyzed for the 545 NAFLD patients using multiple linear regression adjusted for sex and age.**

We selected quantitative-traits related to NAFLD to analyze, including FGF21, ALT, AST, γ-GT, triglycerides, LDL-C, total cholesterol and fasting glucose (Figures 1–3). The results showed that *KLB* SNPs rs7674434 and rs12152703 had significant associations with ALT (*P* = 0.030 and *P* = 0.041, respectively) and γ-GT (*P* = 0.032 and *P* = 0.024, respectively) levels, while SNP rs7670903 correlated with γ-GT levels (*P* = 0.034) (Table 5). The *KLB* SNPs had no significant associations with AST, but SNPs rs7674434 and rs12152703 had significant associations with the AST/ALT ratio (*P* = 0.024 and *P* = 0.013, respectively) (Table 5). Comparison of the FGF21 levels revealed a significant association with *FGF21* SNP rs499765 (*P* = 0.049) (Figure 2). The minor G-allele of rs7674434 and T-allele of rs12152703 correlated positively with ALT and γ-GT levels, the A-allele of rs7670903 correlated positively with γ-GT levels, and G-allele of rs499765 correlated positively with FGF21 levels. On the other hand, there were no significant associations among triglycerides, cholesterol or LDL-C levels and the SNPs.

To confirm the relation between the SNPs and hepatic inflammation in NAFLD patients, we analyzed the associations between the SNPs and ALT and γ-GT levels in the NAFLD group. The results (Figure 3) indicated that the G-allele of rs7674434 and T-allele of rs12152703 had stronger positive correlations with ALT levels in the NAFLD group (*P* = 0.005 and *P* = 0.008, respectively) than in the other groups. By contrast, there were no significant associations with γ-GT, AST or the AST/ALT ratio in the NAFLD group.

## DISCUSSION

An important finding of our study is the strong association between *KLB* SNP rs7670903 and obesity in a Han population of East China. The association was verified through both quantitative-traits analysis and cohort analysis. Another important result is that in the obese population, the minor G-allele of rs7674434 and T-allele of rs12152703 in *KLB* are risk alleles for NAFLD, but in the non-obese population there is no association between *KLB* and NAFLD. In addition, these two SNPs have significant associations with serum ALT levels. Notably, the association with ALT levels in NAFLD subjects is obviously stronger than in all subjects, which verifies the association between *KLB* variants and the degree of liver inflammation in the NAFLD population. As far as we know, there have been no previous reports of a correlation between NAFLD and *KLB* gene polymorphism.

*KLB* is a protein coding gene located in chromosome 4p14. The gene belongs to the glycoside hydrolase family 1 gene group and is mainly expressed in fat and liver. The dominant structure of KLB protein contains two connected glycoside hydrolase-like regions that bind FGF21 and FGF19. Functioning as a co-receptor, KLB is thought to be the primary high-affinity receptor for FGF21 and FGF19 binding to FGFRs (FGFR1 and FGFR4) to activate intracellular signaling [13]. *FGFR1* and *FGFR*4 are widely expressed in most tissues, but the distribution of *KLB* is more specific. Furthermore, KLB combined with endocrine FGFs forms an activated FGFs-FGFR-KLB complex that is involved in regulating metabolic processes [14, 15]. In that regard, FGF21 is known to play a key role in controlling energy homeostasis [5]. In humans, serum FGF21 levels rise under conditions of starvation or inflammation [1]. In diabetic mouse models, blood glucose and triglyceride levels decline and insulin sensitivity is enhanced after mice are injected with recombinant FGF21 [16]. However, serum FGF21 levels are also elevated in the obese population, which likely also has a higher incidence of NAFLD [17]. Moreover, it appears that obesity can lead to an FGF21-resistant state [18] and that *KLB* expression is reduced in obese mice [19, 8]. This suggests reduced KLB expression maybe be an underlying cause of FGF21 resistance and the development of NAFLD.

*KLB* polymorphism is also reportedly related to coronary artery disease [20] and alcohol consumption [21]. In addition, studies in animals [11, 12] have shown that the AST/ALT ratio is elevated and triglyceride accumulation in the liver is increased in mice lacking *KLB*. Conversely, in obese mice overexpressing KLB, circulating cholesterol levels are reduced and the sensitivity to FGF21 is enhanced. Up to now, there have been no studies on the relation between *KLB* polymorphism and NAFLD in humans.

The locus rs7670903 is located within intron 3 of *KLB*, near the end of exon 3 in the protein coding domain. Two other loci, rs7674434 and rs12152703, are both situated within intron 1 of *KLB*, and between exons 1 and 2 in the protein coding domain. The distance between these two loci is 2410 bp, though the two variants show complete (r^2^ = 1.0) linkage disequilibrium in the Chinese population (grch37.emsebl.org), so these two alleles are completely associated. The functions of these three SNPs are unknown, and there are no previous reports of an association between these SNPs and NAFLD. In our analysis, however, rs7674434 was significantly associated with the development of NAFLD in an obese population and with the degree of liver inflammation in a NAFLD population. Moreover, rs12152703, which, as mentioned, is in complete linkage disequilibrium with rs7674434, exhibits a similar association. This further supports the correlation between *KLB* gene polymorphism and NAFLD. This suggests SNPs rs7674434 and rs12152703 may be useful therapeutic targets in NAFLD patients. In particular, *KLB* becomes a risk gene for NAFLD in obese individuals but not non-obese individuals. This hints that *KLB* may be an important risk gene for NAFLD under the environmental pressure of obesity.

NAFLD is a disease with high morbidity and without effective treatments. Recombinant FGF21 is thought to be a promising treatment option for hepatocellular carcinoma (HCC) [14] and NAFLD [22]. However, conditions such as obesity may lead to FGF21 resistance, and the level of *KLB* expression may modulate the response to FGF21 [8]. The *KLB* gene polymorphism is associated with expression of KLB protein suggests *KLB* polymorphism is involved in energy metabolism and the pathogenesis of NAFLD. This makes *KLB* SNPs a potential target for treatment of NAFLD.

There are several limitations to our study. First, we studied patients from a single center, so the results may not represent the entire Chinese population. Second, a significant association between *KLB* and AST levels was not been found in the study. Similarly, we did not find a significant association between *KLB* and the AST/ALT ratio in the NAFLD group. This may be because the number of NAFLD patients, particularly those with severe NAFLD, was too small. Future studies should include additional clinical centers to enlarge the sample size to further test the results of this study. Finally, although associations between three *KLB* SNPs and NAFLD were detected, not all *KLB* SNPs were analyzed. In future studies, we will analyze the other *KLB* SNPs and assess expression of KLB protein in order to identify potential gene targets related to diagnosis and treatment of NAFLD.

## CONCLUSIONS

In the Han Chinese, *KLB* is strongly associated with obesity, the development of NAFLD in the obese population, and hepatic inflammation in NAFLD patients. This means that *KLB* is not only involved in normal energy metabolism but also influences hepatic injury in NAFLD. And because FGF21 depends on KLB as co-receptor to exert its biological effects, *KLB* gene polymorphism may mediate FGF21 resistance to NAFLD treatment.

## MATERIALS AND METHODS

### Study population

From January 2018 to October 2018, a total of 1688 subjects were recruited from the Department of Physical Examination Center, the Affiliated Hospital of Xuzhou Medical University, China (see Supplementary Table 1 for details about the recruitment). Included populations were unrelated and ethnically Han Chinese aged 18-80 years. Exclusion criteria were: 1) history of significant alcohol consumption (>210 g of pure alcohol per week in men or >140 g of pure alcohol per week in women for >1 year); 2) history of long-term medication (>1 week); and 3) infection with hepatitis B or hepatitis C virus. The subjects were first divided into NAFLD (n = 545) and non-NAFLD (n = 1143) groups based on the results of liver ultrasonography. The ultrasonographic manifestation of NAFLD was more enhanced echotexture in liver than kidneys, obscure vascular structures, shallow echo intensification, and deep attenuation of the liver. The subjects were then further divided into obese and non-obese groups. The WHO criteria of obesity in Asian populations is a BMI ≥ 25. There were 584 obese individuals among the subjects. Ultimately, then, the subjects were divided into four groups: non-obese without NAFLD, obese without NAFLD, non-obese with NAFLD and obese with NAFLD. All participants gave their informed consent, and the study protocol was approved by the ethics committee of the Affiliated Hospital of Xuzhou Medical University (XYFY2019-KL012).

### Laboratory measurements

The experimental indexes were measured under fasting conditions in the central laboratory of the Affiliated Hospital of Xuzhou Medical University. Measured were ALT, AST, γ-GT, triglycerides, cholesterol, LDL-C and fasting glucose. In addition, venous blood was collected and centrifuged at 5,000 × g for 10 min, after which the serum was stored at −80°C. Serum FGF21 levels were measured using a Human FGF-21 Simple Step ELISA Kit (ab222506, Abcam, Cambridge, United Kingdom).

### SNP selection

SNPs in *FGF21*, *KLB* and *FGFR* possibly associated with nutrient intake and metabolism were selected for detection. SNPs in *IZUMO1*, *FUT1* and *FGF21* on chromosome 19q13 were reported to influence components of caloric intake in a genome-wide meta-analysis of European population-based cohorts [10] and potentially influenced expression of FGF21 protein [9]. The impacts of SNPs in *FGFR1* and *FGFR2* on obesity and on serum LDL-C levels have been reported in multiple European cohorts [23, 24]. Several studies suggest that SNPs in *KLB* localized on human chromosome 4p14 are probably related to alcohol drinking and HCC progression [21, 25], but the association of *KLB* SNPs with nutrient metabolism were not verified. We selected SNPs with a high frequency (>10%) of the minor allele.

Genomic DNA was isolated from the whole blood using a Tianamp DNA kit (Tiangene, Biotech, Beijing, China) following the manufacturer’s instructions. It was then quantified using a UV spectrophotometer. The locus-specific PCR primers were designed by ASSAY DESIGH SUITE V2.0 (Table 1). The specific amplification was performed using the Sequenom MassARRAY iPLEX platform (Sequenom CapitalBio Corporation, Beijing, China). Masses were detected using a MALDI-TOF mass spectrometer, and the obtained data were analyzed using MassARRAY Typer 4.0 software.

### Statistical analysis

The baseline characteristics of the samples were compared between the groups using the nonparametric Kruskal-Wallis test. We used Plink 1.9 [26] software to control the quality of the genotyping results and carry out association analyses. The quality control included the loss frequency of genotyping, MAF and HWE tests. The genotype-phenotype association analyses were performed using three covariate-adjusted models (dominant, recessive and additive) with age and sex as random effects. Quantitative-traits association analyses were used to assess the interaction between factors for NAFLD and the genotypes of each SNP through multiple linear regression analyses in all subjects. In addition, we analyzed the quantitative-traits association between the biochemistry indexes and the allelic variants in the NAFLD group. Values of *P* < 0.05 considered significant, with smaller *P*-values indicating stronger degrees of association.

## Supplementary Material

Supplementary Table
