# Supplementary figures and images for: KLB gene polymorphism is associated with obesity and non-alcoholic fatty liver disease in the Han Chinese
Source: Aging (Albany NY). 2019 Sep 23;11(18):7847–58. doi: 10.18632/aging.102293 (PMC6781984; doi:10.18632/aging.102293)

## SUPPLEMENTARY TABLE

Supplementary Table 1. Flow chart of the protocol for subject recruitment.

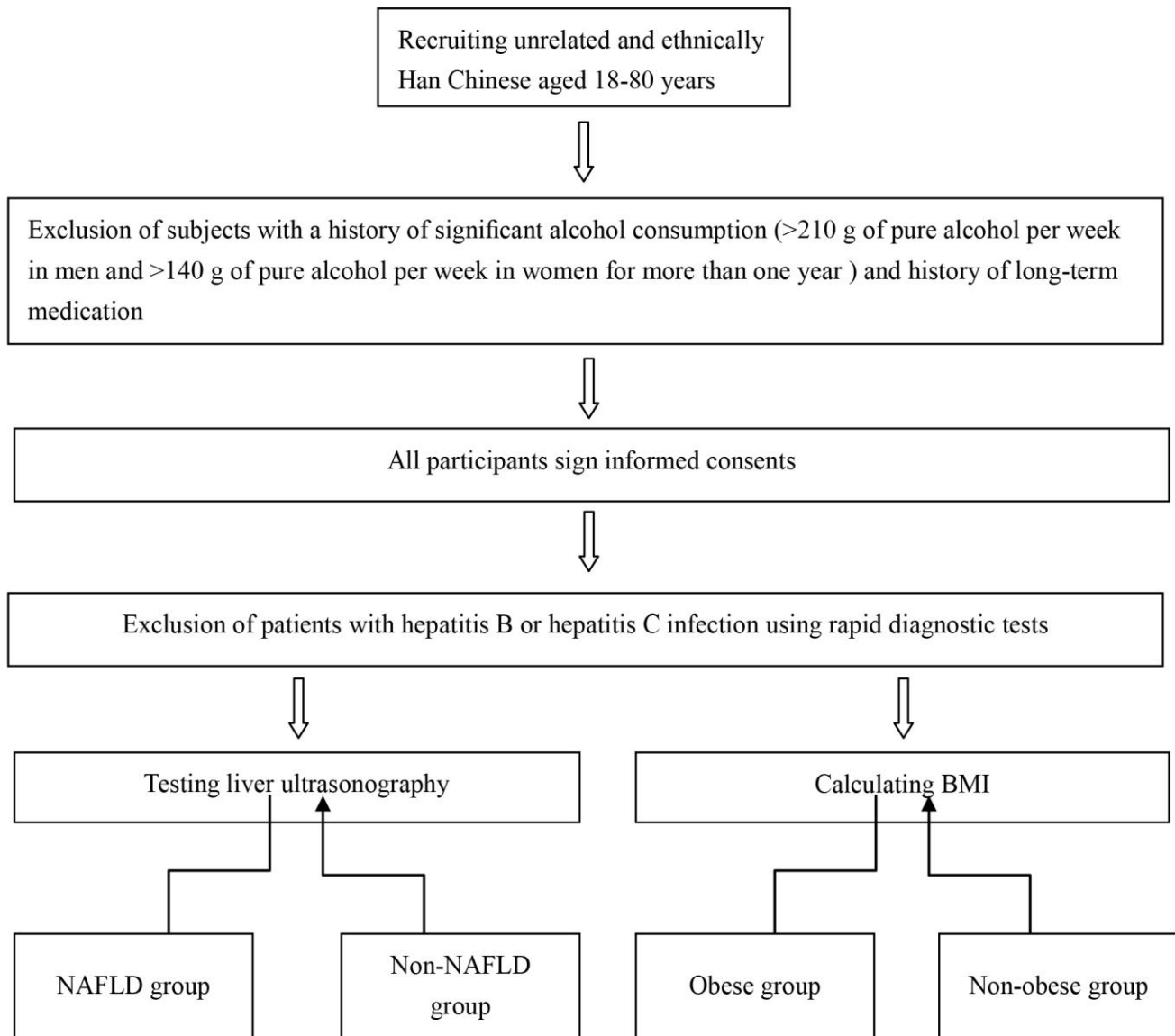

Supplement: Supplementary Table [file aging-11-102293-s001.pdf]
